# Improvement of the Stability and Activity of an LPMO Through Rational Disulfide Bonds Design

**DOI:** 10.3389/fbioe.2021.815990

**Published:** 2022-01-17

**Authors:** Xiaoli Zhou, Zhiqiang Xu, Yueqiu Li, Jia He, Honghui Zhu

**Affiliations:** Key Laboratory of Agricultural Microbiomics and Precision Application - Ministry of Agriculture and Rural Affairs, Guangdong Provincial Key Laboratory of Microbial Culture Collection and Application, State Key Laboratory of Applied Microbiology Southern China, Guangdong Microbial Culture Collection Center (GDMCC), Institute of Microbiology, Guangdong Academy of Sciences, Guangzhou, China

**Keywords:** LPMOs, enzyme engineering, stability, activity, disulfide bonds

## Abstract

Lytic polysaccharide monooxygenases (LPMOs) oxidatively break down the glycosidic bonds of crystalline polysaccharides, significantly improving the saccharification efficiency of recalcitrant biomass, and have broad application prospects in industry. To meet the needs of industrial applications, enzyme engineering is needed to improve the catalytic performance of LPMOs such as enzyme activity and stability. In this study, we engineered the chitin-active CjLPMO10A from *Cellvibrio japonicus* through a rational disulfide bonds design. Compared with the wild-type, the variant M1 (N78C/H116C) exhibited a 3-fold increase in half-life at 60°C, a 3.5°C higher *T*_*50*_^*15*^, and a 7°C rise in the apparent *Tm*. Furthermore, the resistance of M1 to chemical denaturation was significantly improved. Most importantly, the introduction of the disulfide bond improved the thermal and chemical stability of the enzyme without causing damage to catalytic activity, and M1 showed 1.5 times the specific activity of the wild-type. Our study shows that the stability and activity of LPMOs could be improved simultaneously by selecting suitable engineering sites reasonably, thereby improving the industrial adaptability of the enzymes, which is of great significance for applications.

## Introduction

Lytic polysaccharide monooxygenases (LPMOs), the copper-dependent enzymes, catalyze the oxidative cleavage of the glycosidic bonds within crystalline polysaccharides such as cellulose and chitin and provide more binding sites for glycoside hydrolases, thus significantly improving the degradation efficiency of polysaccharides through cooperation with glycoside hydrolases (Vaaje-Kolstad et al., 2010; Eibinger et al., 2014; Hemsworth et al., 2015). The unique enzymatic activity endows LPMOs the broad potentials in industrial application, in particular in biomass-based industries, like second-generation biorefinery (Hemsworth et al., 2015; Barbosa et al., 2020). However, the inhered defects in both the low enzyme activity and stability have prevented the native LPMOs from industrial applications well; consequently, enzyme engineerings are necessary.

LPMOs are characterized with a conserved β sandwich core structure consisting of seven to nine β-strands. The loops connecting these β-strands constitute the ‘flat’ substrate-binding surface, which provides a structural basis for LPMOs to act on crystalline polysaccharides (Hemsworth et al., 2013a; Vaaje-Kolstad et al., 2017; Tandrup et al., 2018). The catalytic center, ‘histidine-brace’, is consisting of the N-terminal histidine and another conserved histidine in coordinating a copper ion, and is located on the substrate-binding surface (Hemsworth et al., 2013b; Ipsen et al., 2021). Studies have indicated that the amino acid composition and topological features of the substrate-binding surface are intimately related to the substrate selectivity and oxidation regioselectivity of LPMOs (Frandsen and Lo Leggio, 2016; Zhou and Zhu, 2020). Guo et al. (2020) obtained an enzyme activity increased variant of a cellulose-active fungal LPMO, MtC1LPMO, by a site-directed mutation on the L2 loop, which provides an example of the activity improvement in LPMOs. In addition, enzyme stability is another limitation of using LPMOs in industries, where the enzyme proteins not only work under harsh conditions but also are expected to last active. To achieve this goal, Lo Leggio et al. (2018) successfully obtained the stability increased AfAA9, an AA9 fungal LPMO from *Aspergillus fumigatus*, by substituting an aspartic acid to serine so changing the unfavorable charge interactions.

Theoretically, the rigidity and flexibility of a protein molecule can indicate its thermostability to a certain extent; and to improve the thermostability of a protein one can reduce its flexibility or increase the rigidity (Yu and Huang, 2014; Sun et al., 2019). However, it must be cautious to increase the thermostability of LPMOs simply via reducing the flexibility, because the substrate-binding surfaces in these proteins are composed of multi-flexible loops as well as constitute a large part of the molecular surfaces. Therefore, residue substitution in this region would take a big risk to damage the enzymatic activity.

In this study, we used CjLPMO10A, a chitin-active LPMO from *Cellvibrio japonicus* (Forsberg et al., 2016), as the model enzyme to explore an approach appropriate for improving the stability of LPMOs. We first performed the B-factor analysis and amino acids coevolution analysis, and selected the highly flexible loops away from the substrate-binding surface and unrelated to the catalytic function of CjLPMO10A as the modifying sites. Through site-directed mutations, a disulfide bond was designed in the flexible loops. The variant (N78C/H116C) was successfully produced in *Escherichia coli*, which not only showed significantly improved thermal and chemical stability than the wild-type but also showed 1.5 times the enzyme activity of the wild-type. This study provides a strategy and experimental basis in enzyme engineering of LPMOs toward industrial applications.

## Materials and Methods

### Cloning

The gene of the LPMO domain of CjLPMO10A (GenBank: ACE83992.1) and the variants M1 and M2 were codon-optimized and synthesized by Sangon Biotech (Sangon Biotech Co., Ltd., Shanghai, China). Then the genes were amplified by PCR with primers cjlpmo10a-F and cjlpmo10a-R (Supplementary Table S3). The vector pET22b was linearized by PCR with primers 22b-f and 22b-r (Supplementary Table S3). Each gene was cloned into the linearized pET22b, generating the recombinant expression plasmids pET22b-cjlpmo10a, pET22b-m1, and pET22b-m2 using the In-Fusion HD cloning kit (Takara Biomedical Technology Co., Ltd., Beijing). The construct was transformed into *E. coli* BL21 (DE3) to express the protein in periplasmic space as described (Zhou et al., 2020).

### Protein Expression and Purification

The resultant *E. coli* BL21 (DE3) cells were cultured at 37°C and 220 rpm in Luria-Bertani (LB) medium containing ampicillin (100 μg/ ml) and chloramphenicol (35 μg/ ml) until the OD600 was approximately 0.6. 0.5 mM isopropyl-β-D-thiogalactopyranoside (IPTG) was added and cultured at 22°C with 200 rpm shaking overnight to induce protein expression. Cells were harvested and the periplasmic proteins were extracted using an osmotic shock method (Manoil and Beckwith, 1986). Then the protein was purified by nickel-chelating chromatography and concentrated using Amicon ultracentrifugal filters (Millipore) with a molecular mass cutoff of 10 kDa. The purified protein was saturated with copper as described previously (Forsberg et al., 2014).

### Enzyme Activity Assays

The reaction system contained 10 mg/ ml shrimp shell chitin (sigma), 50 mM Tris-HCl (pH 7.8), 0.1 mg/ ml enzyme protein, and 1 mM ascorbic acid. The mixture was incubated at 37°C with shaking at 1,000 rpm for 72 h and terminated by heating at 100°C for 10 min. The supernatant was separated from the insoluble substrate particles by filtration using a 0.22 μm filter and analyzed by MALDI-TOF MS as described (Vaaje-Kolstad et al., 2010). Briefly, the samples were analyzed with an UltrafleXtreme MALDI-TOF/TOF instrument (Bruker Daltonics GmbH, Bremen, Germany) using a 2,5-DHB matrix, in positive acquisition mode. All spectra were obtained using the reflectron mode with an acceleration voltage of 20 kV, a reflector voltage of 21.1 kV. The acquisition range used was from *m/z* 500 to 3,000.

The enzyme activity assays using the substrate 2,6-DMP were performed as previously described (Breslmayr et al., 2018). The reaction system contained 50 mM Tris-HCl buffer at pH 7.8, 100 μM H_2_O_2_, 1.0 mM 2,6-DMP, and the reaction time was 300 s. One unit activity was defined as the formation of 1 μmol coerulignone per min under reaction conditions.

The reaction system of the synergy experiments for CjLPMO10A^cd^ or M1 with chitinase contained 10 mg/ ml shrimp shell chitin in 50 mM ammonium acetate buffer (pH 6.2) containing 1 mM ascorbic acid. The protein concentration of CjLPMO10A^cd^ or M1 in the reaction system was 0.25 mg/ ml. The protein concentration of chitinase (Shanghai yuanye Bio-Technology Co., Ltd) was 0.6 mg/ ml. The reaction mixtures were incubated at 37°C with constant shaking at 1,000 rpm in a thermomixer. The amount of released reducing sugar was analyzed using the DNS method.

### Thermal Inactivation

The enzymes (0.1 mg/ ml) were incubated at 60°C and sampled at different intervals to measure the residual activities at 30°C. The average thermal inactivation rate constants (*k*_*inact*_) were calculated from the plots of ln (residual activity) versus time. The time required for the residual activity to be reduced to half (*t*_*1/2*_) of the enzymes was calculated by the equation: *t*_*1/2*_ = ln2/*k*_*inact*_.

The *T*_*50*_^*15*^ value was assayed as previously described (Xie et al., 2014). Briefly, proteins (0.1 mg/ ml in 50 mM Tris-HCl, pH 7.8) were heated at different temperatures for 15 min, and the residual activities were measured at 30°C. The residual activity was plotted against temperature and fitted with a sigmoidal curve using Origin 8.0. The *T*_*50*_^*15*^ value is the temperature at which enzyme activity is reduced to 50% after a 15 min heat treatment.

### Differential Scanning Fluorimetry

The apparent melting temperature of the enzymes was measured via differential scanning fluorimetry (DSF) in a QuantStudio™ 5 System, using the Protein Thermal Shift™ Dye Kit (ThermoFisher). The protein concentrations were 0.2 mg/ ml, and the reaction system was prepared according to the manual of the kit. The temperature was increased from 25 to 99°C with 0.05°C/s increment. The experiment was done in triplicate, and the fluorescence intensity data were fitted to a Boltzmann sigmoidal curve using Origin 8.0 to determine the apparent melting temperature (*Tm*).

### Chemical Stability

The protein (0.03 mg/ ml) was incubated with a set of concentrations of guanidine hydrochloride (Gdn-HCl) for 24 h at 25°C. The protein unfolding was monitored by an FS5 Spectrofluorometer (Edinburgh Instruments Ltd., Livingston, United Kingdom) at 25°C. The fluorescence spectrum was detected in the range of 300–400 nm, with the excitation wavelength of 280 nm and the light diameter of 1 cm. The maximum emission wavelength was plotted against the concentration of guanidine hydrochloride, and the unfolding curve was fitted to the three-state or four-state equilibrium model to obtain the Cm value (Dignam et al., 2001; Yang et al., 2011).

For residual activity assays, the protein (0.1 mg/ ml) was incubated with a set of concentrations of Gdn-HCl for 24 h at 25°C, and the residual activity was measured using 2,6-DMP as substrate. The system for measuring enzyme activity contained the same concentration of Gdn-HCl as the incubation system.

### Homology Modeling and Molecular Dynamic Simulations

The model structures of the variants were created by the SWISS-MODEL (https://swissmodel.expasy.org/) (Waterhouse et al., 2018) using the crystal structure of CjLPMO10A^cd^ (PDB ID: 5FJQ) as the template. Verify_3D (Luthy et al., 1992) and PROCHECK (Laskowski et al., 1996) were used to assess the qualities of the 3D models. The MD simulations were performed using Gromacs v4.5.5 (Pronk et al., 2013), with the Gromacs 96 (54a7) force field. The crystal structure of CjLPMO10A^cd^ and the model structures of the variants were solvated with a three-point water model in a cubic box. Na^+^ and Cl^−^ ions were added to neutralize the charges in the system. Then, a steepest descent energy minimization was performed, followed by a 100 ps NVT and a 100 ps NPT equilibration at 300 K, and 100 ns MD simulations were performed at 300 K. All bond lengths were constrained using the LINCS (LINear Constraint Solver) algorithm (Hess et al., 1997). The cut-off value for van der walls interactions was set at 1 nm, and electrostatic interactions were calculated using a PME (Particle Mesh Ewald) algorithm (Darden et al., 1993).

### Amino Acid Coevolution Analysis

The multiple aligned sequences of LPMO10s were generated from Pfam database (PF03067, a total of 4173 sequences as of July 30, 2021) (Mistry et al., 2021). Then the multiple aligned sequences were uploaded to the Leri web server (https://kornmann.bioch.ox.ac.uk/leri/services/ecs.html) (Cheung et al., 2021) to calculate the evolutionary residues, using the default parameters.

## Results

### Rational Design of Disulfide Bonds in CjLPMO10A^cd^

We hypothesized that the introduction of disulfide bonds in the highly flexible area unrelated to the catalytic function will increase the rigidity of the LPMO molecule, thus improving its stability without destroying the activity of the enzyme. As a proof of concept, CjLPMO10A was engineered to improve its stability through the introduction of disulfide bonds. CjLPMO10A is a tri-modular enzyme, and in addition to the LPMO module, it contains a CBM5 (carbohydrate-binding module) and a CBM73 module (Forsberg et al., 2016). In this study, we used its LPMO module for modification assigned as CjLPMO10A^cd^ (residues 37–216), to preclude the possible influences of CBM domains (residues 217–397). In the following experiments, the amino acid numbers of CjLPMO10A^cd^ were renumbered by starting with the first catalytic Histidine 1.

**FIGURE 1:**
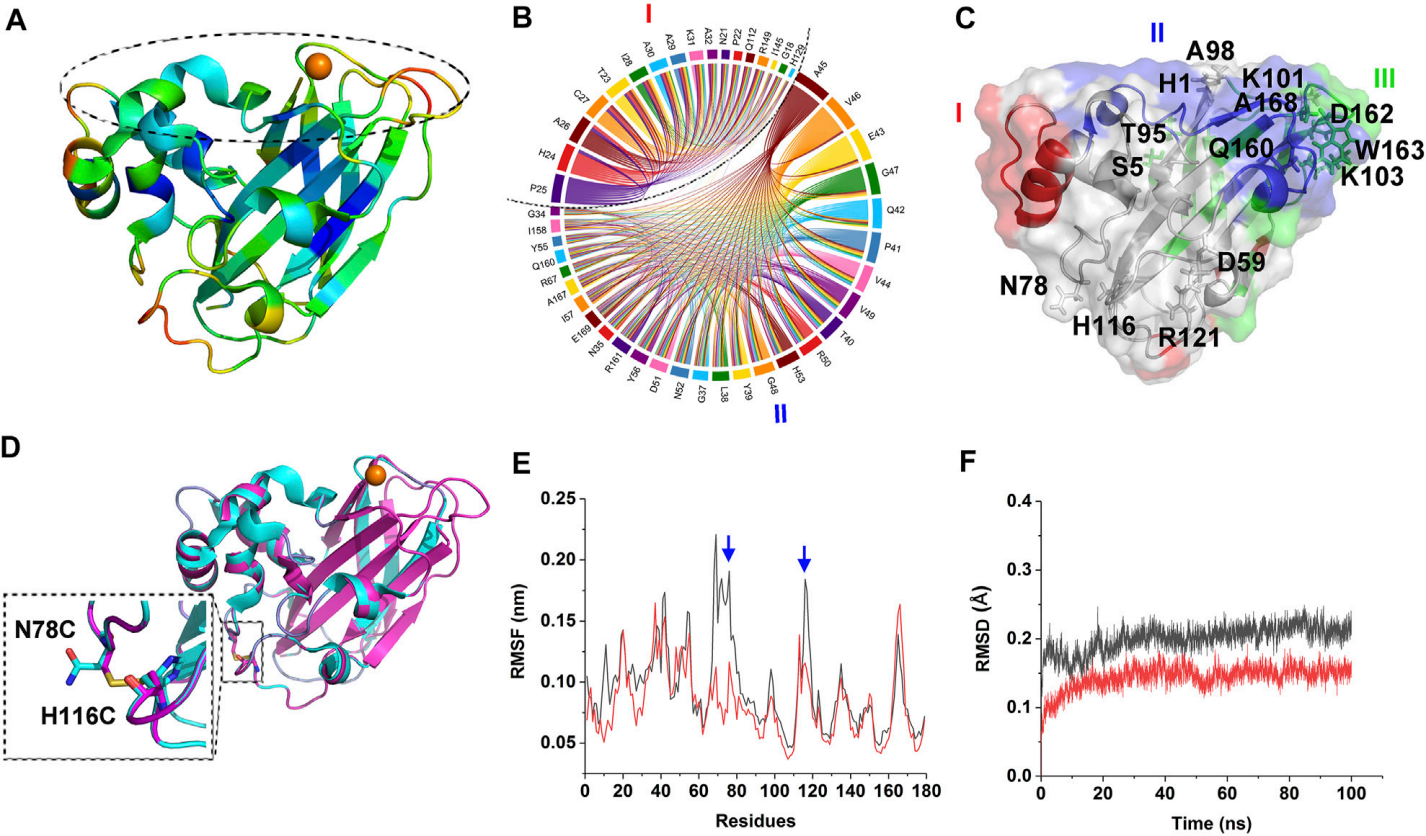
Design of the mutant. **(A)** Structure of CjLPMO10A^cd^. The color from blue to red indicates the B-factor is from low to high. The dashed circle indicates the substrate-binding surface. **(B)** The top two coevolutionary groups from amino acid coevolution analysis of LPMO10s. **(C)** The three coevolutionary residues groups are mapped to the tertiary structure of CjLPMO10A^cd^ (PDB ID: 5FJQ), colored by red, blue and green, respectively. The sites suggested by B-factor and DbD2 analysis are shown in sticks and labeled. **(D)** Superposition of the model structure of M1 (N78C/H116C) and the structure of CjLPMO10A^cd^. Cyan, CjLPMO10A^cd^; magenta, M1. The enlarged dashed box shows the introduced disulfide bond. The mutated sites are labeled and shown in sticks. **(E)** and **(F)** RMSF and RMSD of the 100 ns MD simulations of CjLPMO10A^cd^ (black) and M1 (red). The blue arrows indicate the mutated sites.

B-factor analysis was firstly performed on CjLPMO10A^cd^ to select the engineering sites, which indicated that the β strands in the core structure might have the highest rigidity among the other regions; while loops 31–32, 113–118, 135–138, and 162–167 could be the most flexible regions and followed by loops 20–25, 76–78, 84–87, and some scattered residues such as Arg121, Glu43, Lys101, Ser6, Gln126, Asn143, Glu91, Lys103, Ala98, Ala99, Ser5, His53, Gly48, and His1 (Figure 1A). Among them, loops 31–32, 135–138, 164–167, and residues His1, Ala99, Lys101 are the key elements constituting the substrate-binding surface and catalytic center of CjLPMO10A^cd^. Therefore, one must be cautious to manipulate a modification in these regions.

It is generally accepted that the protein sequences are the results of natural selection during the long-term evolution process, in which, when some sites of a protein are mutated, and other functionally related sites may be concomitantly mutated to maintain the structural and functional stability, namely coevolution (Suel et al., 2003). It is worth noting that the coevolved residues are not always those exhibit a direct interaction, even they could be distant in the tertiary structure. Therefore, we hold the opinion that in the engineering of LPMOs, not only residues directly related to substrate binding and catalysis but also their coevolved residues, need to be avoided from being destroyed. To find the appropriate engineering sites and exclude those sites where mutations may cause damage to the catalytic function we performed amino acids coevolution analysis on LPMO10s (Pfam: PF03067). The analysis indicated three groups of coevolutionary residues in the protein (Figures 1B,C; Supplementary Table S1), in which Groups I and II encompass most residues of the L2 loop at the substrate-binding surface. Group I also contains the residues on the other side of the molecule that are far away from the substrate-binding surface, such as Gln112, Ile145, and Arg149. Group III mainly includes the residues of the β strands constituting the core structure and the residues of short loops which connect the β strands and constitute parts of the substrate-binding surface. These results indicated that though most of the coevolutionary residues are located on the substrate-binding surface, some are located far away from the substrate-binding surface, and therefore, the damage of these coevolutionary residues should be avoided during enzyme engineering.

We then, using the Disulfide by Design 2 (Craig and Dombkowski, 2013), analyzed the sites that disulfide bonds could be formed in the CjLPMO10A^cd^ structure (Supplementary Table S2). The top quarter of the sites with the highest sum of B-factors are shown in Figure 1C. In addition to the catalytic His1, the residues Asp162, Ser5, Thr95, Trp163, Ala168, and Lys103 are involved in the coevolutionary Group III and the Gln160 involved in the coevolutionary Group II should also be avoided to be mutated. Integrating the analysis of the B-factor, the possible disulfide bond sites, and the coevolutionary residues, we found that the sites N78-H116 and D59-R121 are located in the flexible loops away from the substrate-binding surface and not involved in the coevolutionary residues groups (Figures 1A,C). Consequently, the four residues were substituted by cysteine to introduce disulfide bonds in CjLPMO10A^cd^, and the variants CjLPMO10A^cd^_N78C/H116C and CjLPMO10A^cd^_D59C/R121C were assigned as M1 and M2, respectively. The structural modeling of the variants not only showed a disulfide bond between the mutated residues, but also the mutated proteins folded into a wild-type structure without significant changes in the overall structure (Figure 1D; Supplementary Figure S1A). Next, a-100 ns molecular dynamics (MD) simulations were performed on the wild-type and the variants to predict whether residue substitutions change the conformational stability of the enzyme. The results showed a significantly lower RMSF (root mean square fluctuation) value of the mutation sites and adjacent residues than that of the wild-type (Figure 1E; Supplementary Figure S1B), indicating reduced flexibility in these regions. Moreover, decreased RMSD (root mean square deviation) value also indicated an increase in the overall rigidity of the variants compared with the wild-type (Figure 1F; Supplementary Figure S1C).

### The Introduction of a Disulfide Bond Increased the Enzyme Activity of CjLPMO10A^cd^

**FIGURE 2:**
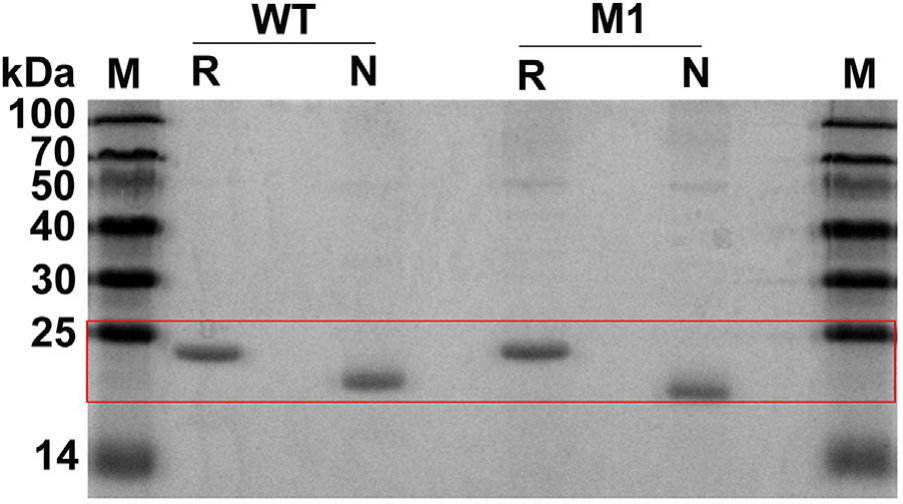
SDS-PAGE analysis of the purified CjLPMO10A^cd^ (WT) and the mutant M1. M, molecular weight marker; R, reducing conditions with DTT; N, non-reducing conditions without DTT.

Wild-type CjLPMO10A^cd^ and the variants were expressed in the periplasmic space of *E. coli* BL21 (DE3) to obtain mature enzyme proteins. Unfortunately, the expression level of M2 was too low to purify enough proteins for biochemical characterization. The purified proteins of CjLPMO10A^cd^ and M1 were analyzed by reducing and non-reducing SDS-PAGE (Figure 2). Under reducing conditions, both the wild-type and M1 showed a band at a molecular weight of ∼21 kDa. The introduction of an additional disulfide bond in M1 will make it fold more tightly than the wild-type, which has two pairs of intramolecular disulfide bonds. The migration distance of M1 under non-reducing conditions was longer than that of the wild-type, indicating that the expected disulfide bond formed well at the mutation sites.

**FIGURE 3:**
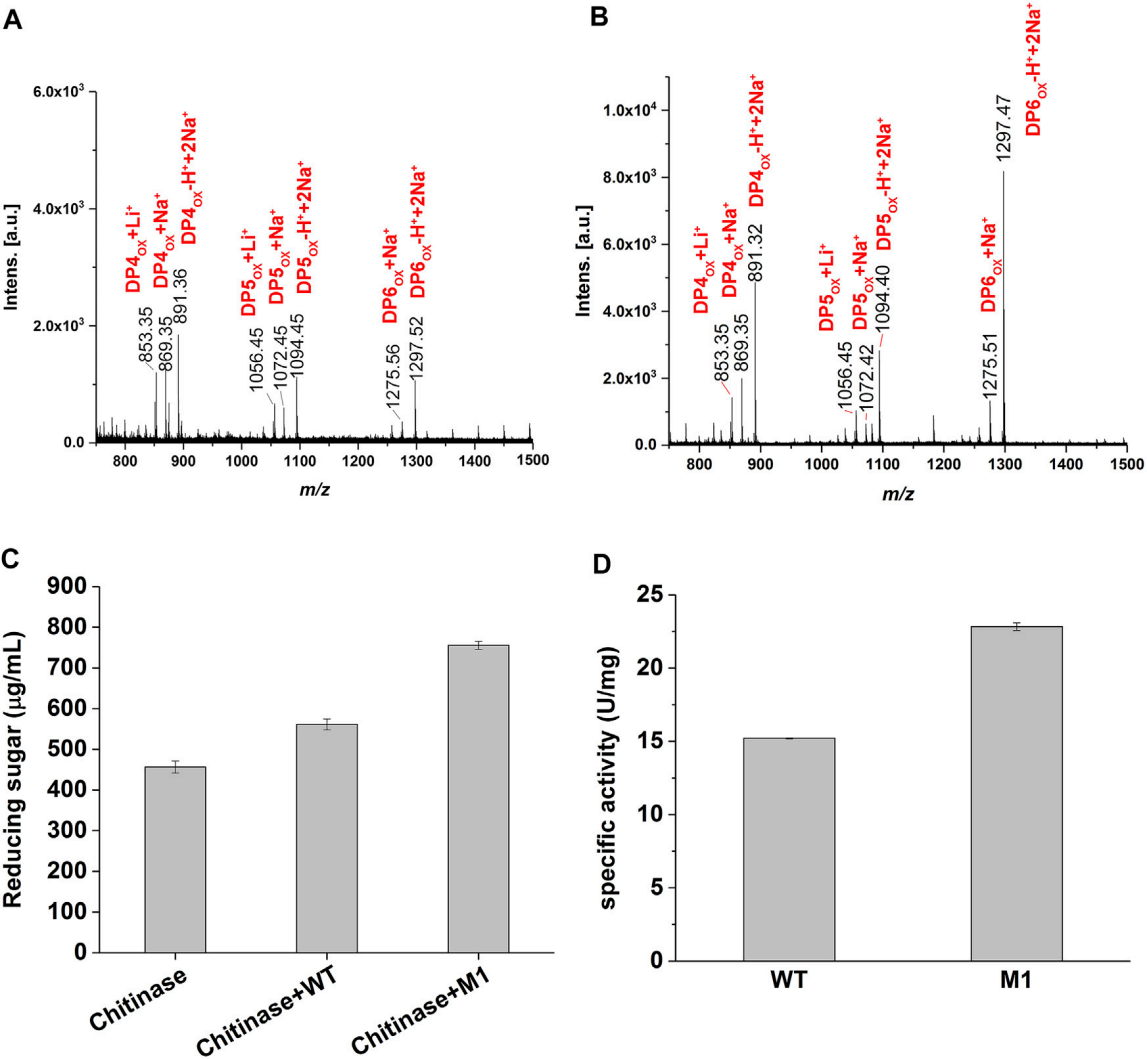
Enzyme activity of CjLPMO10A^cd^ and M1. **(A)** and **(B)** MALDI-TOF MS analysis of the products generated by the wild-type and M1 acting on α-chitin, respectively. DPnox indicates the degree of polymerization (DP) of oxidized chitooligosaccharides. **(C)** Synergistic degradation of α chitin by the wild-type or M1 with chitinase. The reaction mixtures were incubated at 37°C for 40 h, and the released reducing sugars was analyzed using the DNS method. **(D)** The specific activity of the wild-type and M1 measured using 2,6-DMP as substrate.

To confirm that M1 retained the oxidative chitinolytic activity, α chitin extracted from the shrimp shell was used as the enzymatic substrate. After incubation at 37°C for 72 h, the products were subjected to MALDI-TOF MS analysis. The results determined similar predominant compounds from the wild-type and M1, those possess molecular ion peaks of *m/z* 853.3, *m/z* 869.3, *m/z* 891.3, *m/z* 1,056.4, *m/z* 1,072.4, *m/z* 1,094.4, *m/z* 1,275.5, and *m/z* 1,297.5 (Figures 3A,B) and could correspond to C1-oxidized DP4ox, DP5ox, and DP6ox chitooligosaccharides, respectively. This determined that the variant M1 has retained the activity of oxidative cleavage of α chitin as the wild-type enzyme, namely the introduction of a disulfide bond at the selected sites would not damage the catalytic function.

When synergyze with chitinase to degrade shrimp shell chitin, the reducing sugar produced in the variant M1 group was significantly higher than that in the wild-type group for 40 h (Figure 3C). Using 2,6-dimethoxyphenol (2,6-DMP) and H_2_O_2_ as the substrates as described previously (Breslmayr et al., 2018), the specific activities of 22.8 U/ mg and 15.2 U/ mg were detected for the variant M1 and the wild-type, respectively (Figure 3D), namely, 1.5-fold higher activity was achieved through the introduction of a disulfide bond in CjLPMO10A^cd^. Together, these results showed that the mutations did not impair the catalytic function of the enzyme, but increased its activity.

### The Introduction of a Disulfide Bond Increased the Stability of CjLPMO10A^cd^

**FIGURE 4:**
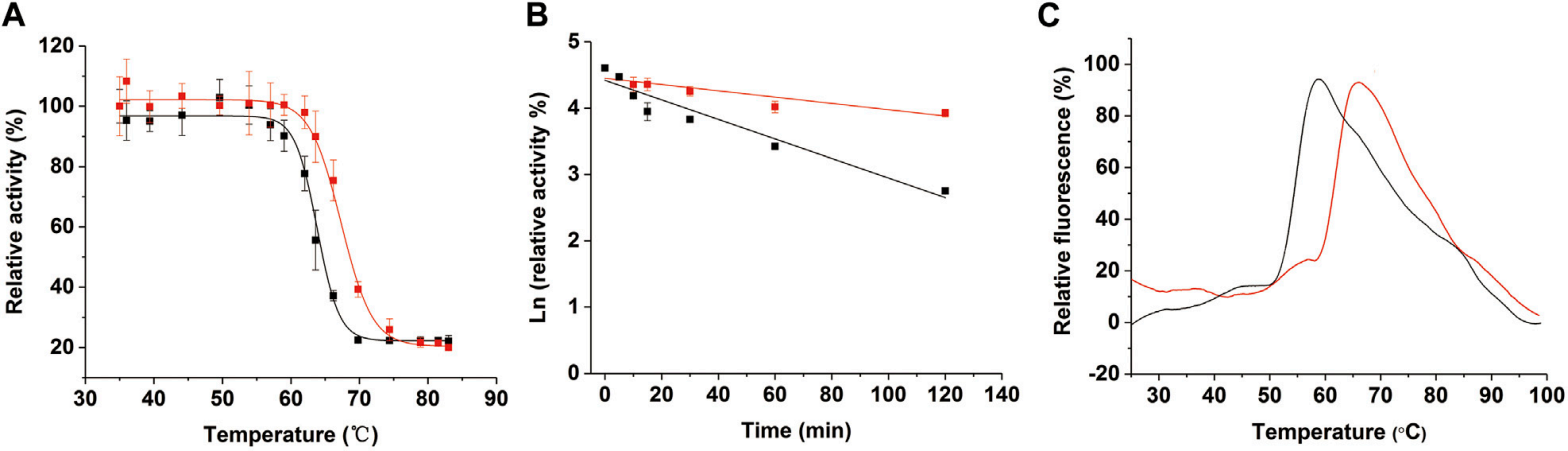
Thermal stability of CjLPMO10A^cd^ (black) and M1 (red). **(A)** Thermal inactivation of CjLPMO10A^cd^ and M1 incubated at different temperatures for 15 min. Proteins in 50 mM Tris-HCl buffer, pH 7.8 were incubated at various temperatures for 15 min and assayed for residual activity at 30°C. The activity of the samples without incubation was considered to be 100%. **(B)** Thermal inactivation of CjLPMO10A^cd^ and M1 at 60°C. Proteins were incubated at 60°C and sampled at regular intervals to determine residual activity at 30°C. **(C)** Thermal unfolding of CjLPMO10A^cd^ and M1 analyzed by DSF. The protein concentrations were 0.2 mg/ml, and the temperature was increased from 25 to 99°C with 0.05°C/s increment. All experiments were performed in triplicate.

To determine whether the introduction of the disulfide bond improved the stability of CjLPMO10A^cd^, we first compared the thermal stability of the wild-type and M1. After a 15 min incubation in a temperature range from 35–85°C of the purified enzyme proteins, the residual activities were measured using 2,6-DMP as substrate. As shown in Figure 4A, while the wild-type started to lose part of its activity at 57°C, the variant M1 still maintained 100% activity at 62°C. The *T*_*50*_^*15*^ value, the temperature when half of the enzyme activity is retained after 15 min incubation, was calculated to be 63.8°C and 67.3°C for the wild-type and M1, respectively (Table 1). Supportively, the 60°C-thermal inactivation profile showed that the activity of M1 decreased more slowly than the wild-type (Figure 4B). Their half-lives at 60°C were calculated as 47 and 145 min, respectively.

**TABLE 1:** Thermodynamic parameters of CjLPMO10A^cd^ and M1.

Enzyme	*T*_*50*_^*15*^ (°C)	*t*_*1/2*_ at 60°C (min)	*Tm* (°C)	C_m_(M)^a^		
				C_m1_	C_m2_	C_m3_
CjLPMO10A^cd^	63.8	47.1	54.6	1.3	4.2	−
M1	67.3	145.9	61.7	1.6	3.6	5.1

a Concentration of Gdn-HCl at the transition midpoint.

Similar results were obtained from DSF (differential scanning fluorimetry) analysis. As shown in the melting curves (Figure 4C), the temperature at which wild-type and M1 began to unfold was 50°C and 60°C, respectively. The apparent *Tm* value of M1 was 61.7°C, 7.1°C higher than that of the wild-type, which was 54.6°C (Table 1). Collectively, the experimental data determined that the introduction of a disulfide bond improves the thermostability of CjLPMO10A^cd^.

**FIGURE 5:**
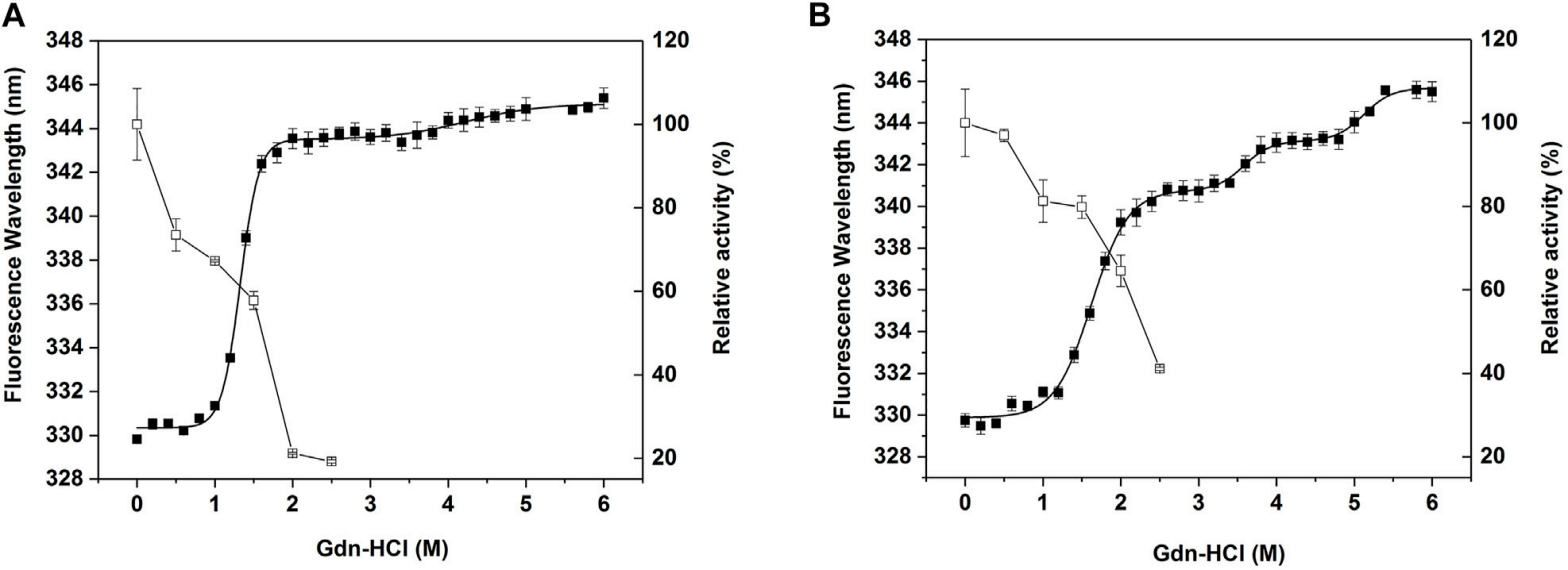
Equilibrium unfolding and chemical inactivation of **(A)** CjLPMO10A^cd^ and **(B)** M1 in guanidine hydrochloride (Gdn-HCl). Enzymes were incubated in different concentrations of Gdn-HCl for 24 h and then assayed for residual activity (□) and fluorescence spectra (■). The residual activity was measured at 30°C, the activity in the absence of Gdn-HCl was considered to be 100%. The fluorescence spectra were recorded at wavelengths between 300 and 400 nm with an excitation wavelength at 280 nm. The maximum emission wavelength was plotted against the concentration of Gdn-HCl, and the curve was fitted to the three-state or four-state equilibrium model. All experiments were performed in triplicate.

To compare the stability of CjLPMO10A^cd^ and M1 against the chemical denaturant, the unfolding curve of the wild-type and M1 were determined in different concentrations of guanidine hydrochloride (Figure 5). The wild-type protein presented a curve that approximated a three-state denaturation model (Figure 5A). Whereas, the redshift of the maximum fluorescence emission wavelength of the wild-type protein in 1.8 M guanidine hydrochloride was close to that in 6 M guanidine hydrochloride (Supplementary Figure S2A), indicating that the protein was almost fully unfolded at this concentration. The Cm value, the concentration of guanidine hydrochloride, for the first step unfolding was 1.3 M (Table 1). The enzyme activity of the wild-type decreased rapidly with the increase of guanidine hydrochloride concentration. After treatment with 2 M guanidine hydrochloride, the residual enzyme activity was only about 20% (Figure 5A). Unlike the wild-type, the unfolding process of M1 appeared an additional stable intermediate transition state, showing a four-state curve (Figure 5B). When the concentration of guanidine hydrochloride was lower than 1.4 M, there was no obvious redshift in the maximum fluorescence emission wavelength (Supplementary Figure S2B), suggesting that the M1 protein was close to the natural folded state. The Cm value of the first unfolding of M1 was 1.6 M, which was 0.3 M higher than that of the wild-type. Correspondingly, the enzyme activity of M1 decreased more slowly than that of the wild-type, and the residual activity after treatment with 2 M guanidine hydrochloride was still about 65% (Figure 5B). These results indicate that the introduction of the disulfide bond improved the stability of CjLPMO10A^cd^ to chemical denaturant.

## Discussion

Enzyme activity and stability are the two most important properties of industrial enzymes that enzyme engineering seeks to improve. Directed evolution and semi-rational design combined with high-throughput screening are effective enzyme engineering methods to modify enzyme activity and stability. Vincent G. H. Eijsink’s lab has developed an MS-based high-throughput screening method for LPMOs enzyme activity screening (Jensen et al., 2019). More screening methods for enzyme engineering of LPMOs still need to be developed.

In rational design, the introduction of disulfide bonds has been successfully used in the engineering of various enzymes to improve their stability (Navone et al., 2021; Wang et al., 2021). Tanghe et al. (2017) obtained the stability improved ScLPMO10C, a cellulose-active LPMO from *Streptomyces coelicolor*, by introducing disulfide bonds into the protein, proving that this strategy is applicable to LPMOs. However, it is difficult to achieve simultaneous improvement in both enzyme activity and stability, and generally, the improvement in one is accompanied by the weakening of the other. Therefore, to improve the stability without destroying the activity, it is necessary to carefully select engineering strategies and sites in rational design. LPMOs are a special kind of enzyme since their most important functional area, the substrate-binding surface composed of flexible loops, occupies a large part of the protein surface, and it is likely to compromise catalytic function when increasing stability by simply reducing the flexibility.

Based on B-factor and amino acid coevolution analysis, we introduced a disulfide bond into the highly flexible loops unrelated to catalytic function to engineer a robust variant, M1. Its thermal stability was enhanced significantly as it showed a 3-fold increase in half-life at 60°C and a 7°C improvement in the apparent *Tm* value. Additionally, the increase in the residual activity and Cm value of unfolding in guanidine hydrochloride also indicated the improvement of its chemical stability. More importantly, the enzyme activity was increased by 1.5 times, which indicates that the mutations did not impair catalytic function, and the increase in the overall stability of the enzyme made it more robust and therefore higher enzyme activity. We also analyzed ScLPMO10C in the aforementioned reference (data not shown), and the results of B-factor and disulfide bond formation sites analysis covered the mutation sites constructed by the authors, such as A143-P183, A52-P61, and S73-A115, etc. Among them, S73 and A115 were not involved in coevolution, while A52 and A143 were involved in the Group I and III coevolution, respectively. In the authors’ results, a certain degree of decrease in enzyme activity caused by mutations at sites A52 and A143 may be related to coevolution. The factors affecting enzyme activity and stability are complex, and more studies are needed to uncover the underlying mechanisms.

In summary, we engineered a disulfide bond into the highly flexible loops away from the substrate-binding surface of CjLPMO10A^cd^. The variant M1 showed 1.5 times the enzyme activity of the wild-type, 3 times the half-life at 60°C, 7°C higher the apparent *Tm* value, and increased resistance to chemical denaturation, indicating improved enzyme activity and stability. Since LPMOs are important enzymes in biomass conversion, the improvement of their enzyme activity, stability, and other properties through enzyme engineering is of great significance for the industrial application of these enzymes.

## Data Availability

The original contributions presented in the study are included in the article/Supplementary Material, further inquiries can be directed to the corresponding author.
