# Supplementary material for: Improvement of the Stability and Activity of an LPMO Through Rational Disulfide Bonds Design
Source: Front Bioeng Biotechnol. 2022 Jan 17;9:815990. doi: 10.3389/fbioe.2021.815990 (PMC8801915; doi:10.3389/fbioe.2021.815990)
Supplement: Supplementary file 1 [file DataSheet1.docx]

Supplementary Material

# Supplementary Figures and Tables

## Supplementary Figures


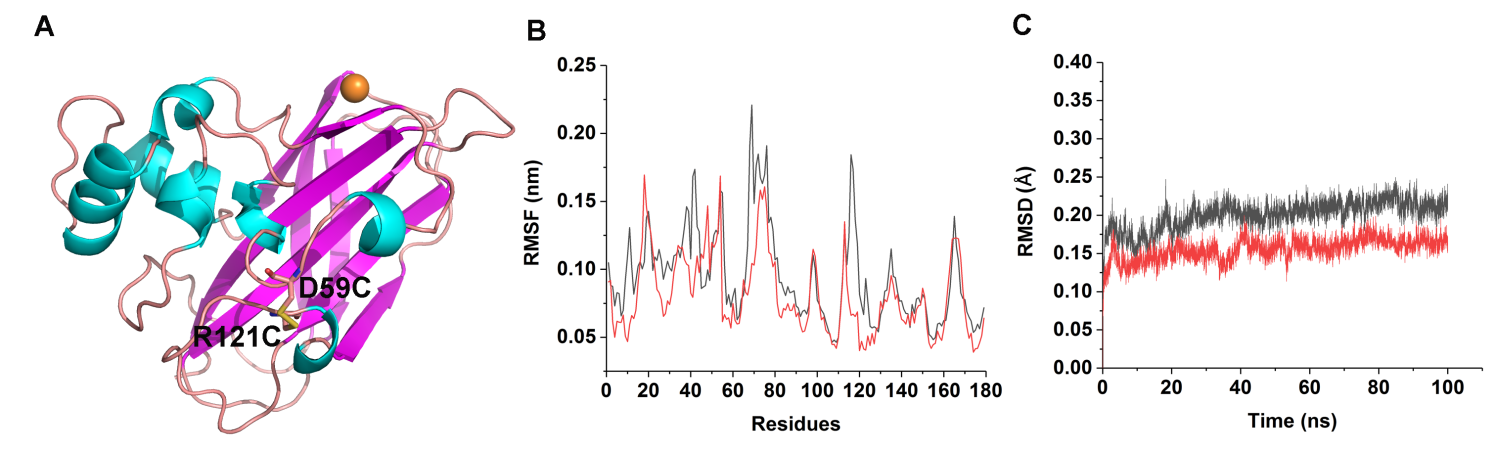


**Supplementary Figure 1.** Model structure and MD simulations of M2 (D59C/R121C). (A) Model structure of M2. The mutated sites are labeled and the introduced disulfide bond is shown in sticks. (B) and (C) RMSF and RMSD of the 100 ns MD simulations of CjLPMO10A^cd^ (black) and M2 (red).


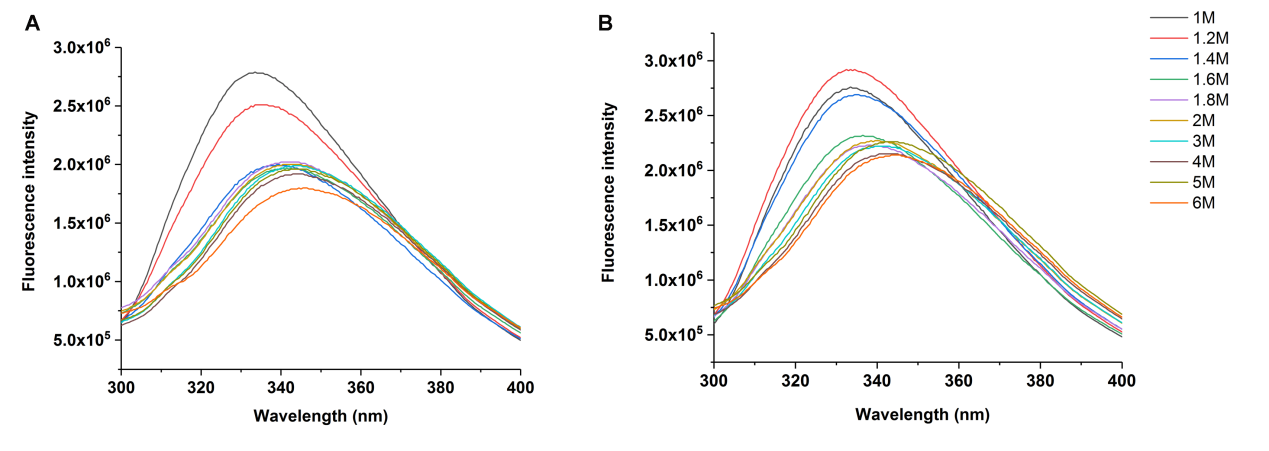


**Supplementary Figure 2.** Fluorescence spectra of the wild-type (A) and M1 (B) in guanidine hydrochloride of different concentrations.

## Supplementary Tables

**Supplementary Table 1** Coevolutionary groups from amino acid coevolution analysis of LPMO10s.

| **Groups** | **Residues^a^** |
| --- | --- |
| I | P25, H24, A26, C27, T23, I28, A30, A29, K31, A32, N21, P22, Q112, R149, I145, G18, H129 |
| II | A45, V46, E43, G47, Q42, P41, V44, V49, T40, R50, H53, G48, Y39, L38, G37, N52, D51, Y56, R161, N35, E169, I57, A167, R67, Q160, Y55, I158, G34 |
| III | G132, P133, S131, A134, Q136, D135, H142, E137, G170, S157, F171, R165, D164, K103, S140, F92, I155, I128, T102, W163, D162, A168, W94, V139, T141, D54, A99, Q126, Y104, D166, T95, S5 |

^a^ The residue numbers refer to the sequence of CjLPMO10A^cd^.

**Supplementary Table 2** Possible disulfide bond formation sites predicted by DbD2.

| **No.** | **Res.1** | **Res.2** | **Sum B factors** | **Bond** | | **No.** | | **Res.1** | **Res.2** | | **Sum B factors** | | **Bond** | | |
| --- | --- | --- | --- | --- | --- | --- | --- | --- | --- | --- | --- | --- | --- | --- | --- |
|  |  |  |  | **χ3** | **Energy (kcal/mol)** |  |  |  |  |  |  |  | **χ3** | **Energy (kcal/mol)** | |
| 1 | Asn78 | His116 | 52.33 | -116.53 | 4.97 | 13 | Pro22 | | | Cys27 | 37.27 | 83.12 | | | 3.03 |
| 2 | Asp59 | Arg121 | 50.94 | 97.91 | 4.7 | 14 | Tyr108 | | | Leu125 | 37.05 | 71.98 | | | 7.79 |
| 3 | Lys101 | Asp162 | 50.62 | 103.47 | 3.45 | 15 | Val4 | | | Ser9 | 36.07 | +126.02 | | | 5.15 |
| 4 | His1 | Ala98 | 45.97 | +85.51 | 4.14 | 16 | Ala82 | | | Asp176 | 35.49 | 94.32 | | | 3.2 |
| 5 | Ser5 | Thr95 | 44.61 | +108.78 | 2.52 | 17 | Ser9 | | | Gln173 | 35.38 | 126.87 | | | 4.37 |
| 6 | Trp163 | Ala168 | 43.41 | 78.53 | 5.35 | 18 | Trp80 | | | Asp176 | 34.76 | 100.9 | | | 2.45 |
| 7 | Lys103 | Gln160 | 43.03 | 126.22 | 6.03 | 19 | Ser64 | | | Leu70 | 34.14 | 82.24 | | | 1.79 |
| 8 | Arg61 | Asn66 | 42.31 | -99.89 | 4.61 | 20 | Cys14 | | | Cys27 | 33.3 | -88.2 | | | 0.76 |
| 9 | Thr110 | Tyr114 | 41.51 | -89.77 | 3.78 | 21 | Phe105 | | | Ser131 | 33.02 | 109.16 | | | 2.94 |
| 10 | Arg161 | Glu169 | 41.45 | 123.2 | 3.82 | 22 | Cys63 | | | Cys174 | 32.87 | -88.8 | | | 0.26 |
| 11 | Pro7 | Thr83 | 40.91 | -86.26 | 2.07 | 23 | Cys63 | | | Met73 | 31.40 | 96.88 | | | 3.48 |
| 12 | Thr102 | Ala134 | 39.70 | 91.12 | 6.19 | 24 | Lys8 | | | Ile12 | 31.14 | 104.25 | | | 4.26 |

**Supplementary Table 3** Primers used in this study.

| **Primer** | **Sequence** |
| --- | --- |
| cjlpmo10a-F | 5’-CTGCCCAGCCGGCGATGGCCCATGGCTACGTTAGCAGCCC-3’ |
| cjlpmo10a-R | 5’-TCAGTGGTGGTGGTGGTGGTGGCCGAAATCAACATCGATGCA-3’ |
| 22b-f | 5’- CACCACCACCACCACCACTGAGATCCGGCTGCTA-3’ |
| 22b-r | 5’- GGCCATCGCCGGCTGGGCAGCGAGGAGCAGCAGA-3’ |
